# Determination of dichlorvos residue levels in vegetables sold in Lusaka, Zambia

**DOI:** 10.11604/pamj.2016.23.113.8211

**Published:** 2016-03-16

**Authors:** Davies Mwazi Sinyangwe, Boniface Mbewe, Gibson Sijumbila

**Affiliations:** 1 Department of Chemistry, University of Zambia, Lusaka, Zambia; 2 Department of Physiological Science, University of Zambia, Lusaka, Zambia

**Keywords:** Vegetables, pesticides, organophosphates

## Abstract

**Introduction:**

Small scale and large scale farmers around Lusaka, the capital city of Zambia grow vegetables using intensive agriculture methods to satisfy the ever increasing demand. To ensure maximum yield they apply various types of pesticides to control pests and diseases that attack these vegetables. Organophosphate pesticides are widely used in agriculture for the control of various insect pests mainly in developing countries. The purpose of the study was to determine the residual levels of the most commonly used organophosphate, 2, 2-Dichlorovinyl dimethyl phosphate, in three commonestvegetables supplied at various markets around Lusaka.

**Methods:**

Samples of 9 bunches of rape, 14 bunches lettuce and 15 rolls cabbage were randomly picked from several study sites around Lusaka. The vegetables were chopped into small pieces which were chemically treated to get methanol extracts. The extracts were then dissolved in an appropriate solvent and using Shimadzu High Performance Liquid Chromatography-Ultra-violet detector (HPLC-UV) levels of 2, 2-Dichlorovinyl dimethyl phosphate were determined.

**Results:**

The analysis showed that the average levels of dichlorvos were significantly above the maximum accepted limit as set by Zambian Food and Drugs Act on vegetables.

**Conclusion:**

Locally grown vegetables from around Lusaka have higher than maximum acceptable limits. This may have implications on human health as the cumulative effect of organophosphates in human body has potential to cause long term health problems.

## Introduction

Pesticides can be defined as chemical substances considered as poisons and used in certain circumstances to kill specifically targeted pests or as chemicals or biological substances used to control pests. They may target pests such as insects, fungus, mollusks, microbes and nematodes which may be vectors of disease of plants resulting in reduced production of vegetables and fruits. Pesticides may be grouped based on target organisms and chemical structure. Based on the target organisms pesticides may be classified insecticide, rodenticides, herbicides, and weedicides [1]. Based on the chemical nature of the pesticide, may be classified as organophosphate (OPP), organochlorides and carbamates.

Vegetables are important ingredient of the human diet for the maintenance of the health and prevention of disease in humans [2]. Vegetable consumption ensures adequate dietary supply of vitamins, minerals, water, and dietary fiber [3]. Vegetables like other crops are invaded by pests and disease like aphids, diamond moths and caterpillars during production and storage leading to reduction of quality and yield. In order to minimize loss and maintain the quality of fruits and vegetables harvested, pesticides are applied in combination with other pest management techniques during cropping to destroy pest and prevent diseases. When applied to vegetable or any other plants small amounts of pesticide residues may remain in the crops or animal feed or environment leading to contamination [4]. The effect of OPP on human health is mainly by virtual of it action on the enzyme acetyl cholinesterase. OPPs exert its effect on pest as well as humans by inhibiting acetyl cholinesterase at nerve endings and nerve junctions [5]. The function of acetyl cholinesterase is to inactivate the neural transmitter, acetylcholine on the nerve junctions and nerve endings. The inactivation is effected by hydrolyzing it to choline and acetic acid or acetyl CoA. Acetylcholine transmits impulses across the nerve junction to effect various biological functions. This enzyme inactivation, leads to acetylcholine accumulation, hyper stimulation of nicotinic and muscarinic receptors, and disrupted neurotransmission [6]. The 2, 2-Dichlorovinyl dimethyl phosphate (dichlorvos) is the most widely used OPP in Zambia. Zambian Food and Drugs Act No. 13 of 1994 defines the maximum residue limit (MRL) as1.0ppm (1 mg/kg) for cabbage, rape and lettuce.

Research has revealed that since 1950, the use of pesticides worldwide has increased and it has been estimated that worldwide about 22 million Kg of pesticides are used annually [7]. This is to be expected as food security particularly in developing countries is very high on the international agenda. Even though pesticides are manufactured under very strict regulations so that there is minimal impact on human health and the environment, serious concerns have been raised about health risks resulting from residues in food [8–11]. By their very nature, most pesticides show a high degree of toxicity because they are intended to kill certain organisms and this creates some risk of harm to humans as well. It is because of this that pesticide use has evoked grave concerns not only of potential effects on human health but also about impacts on wildlife and sensitive ecosystems [12, 13]. Pesticide residues in or on plants may be unavoidable even when pesticides are used in accordance with good agriculture practices but it's the bio accumulative effect in human body that is of concern [14]. Research conducted for the past decade in a number of countries point to the presence of pesticide residues in a number of food items including strawberries, onions, cucumber, lettuce, cabbage, okra, pepper, tomatoes, beans, oranges and lemons [15, 16]. Acute poisoning with an acetyl cholinesterase inhibitor in humans may cause weakness, headache, tightness in chest, blurred vision, salivation, sweating, nausea, vomiting, diarrhea, and abdominal cramps.

Apparently there no measures in place for monitoring and regulating the residues of OPP in vegetables sold in various markets in Lusaka. Evaluating and documenting the levels of dichlorvos in vegetables at the point of sale provides necessary protection for the consumers and provides feedback to the farmers about the safety of their vegetables hence our decision to carry out a study to determine whether people consuming these vegetables are being exposed to higher than acceptable levels of organophosphate residues in vegetables.

## Methods

The study was conducted in Lusaka, the capital city of the Republic of Zambia. Four vegetable growing areas were identified within the vicinity to of Lusaka to the North, South, East and West; and the vegetables were purchased from the markets which were supplied by these farms.

The samples were taken in accordance with the guidelines of the European Union Commission, 1979; which ensured that the samples were taken from the sampling sites at different locations distributed throughout the study sites. Fresh vegetables weighing a minimum of 2kg were collected in polypropylene sealable bags and labeled with a unique sample identity and placed in an iced chest box. A systematic sampling method was used to select the facilities and then simple random sampling method was used to select the vegetables. 15 rolls cabbages, 14 bunches of lettuce and 9 bunches of rape were sampled from the study site. These vegetables were collected from January to June, 2015. The sampling was repeated three times on different days. During the sample collection disposable gloves were used and changed every time before collecting the next sample. Dust from the samples was removed with light brushing, without washing prior to placing them into the collection bags. Samples were double packed, and transported from the field to the Zambia Bureau of Standards (ZABS) Laboratory, Lusaka, Zambia. In the laboratory, samples were placed in the refrigerator at 4°C and analyzed within 7days.

About 1 kg of each vegetable sample was chopped in an electrical chopper and 50 g of chopped sample was taken in an Erlenmeyer flask with sodium chloride (2.5 g), anhydrous sodium sulfate (10 g) and ethyl acetate (60 mL) and mixed using a horizontal shaker for an hour followed by filtration with an ordinary filter paper. Filtered extract was cleaned using 6 g of activated charcoal. The cleaned extract was concentrated on a rotary evaporator and was then dried by bubbling nitrogen gas through it. The dried extract was then re-dissolved in an acetonitrile solvent and final extract of one mL was injected in the liquid chromatograph. The Shimadzu High Performance Liquid Chromatography-UV-VIS spectroscopy was used for measurement of dichlorvos residues.

All the chemicals and reagents that were used were of analytical grade. Pesticides standards were provided by ZABS. Pesticide grade ethyl acetate (HPLC grade) and analytical grade acetone were supplied by HIMEDIA, sodium sulphate (purities greater than 98%) was purchased from GLASSWORLD, and while sodium chloride was supplied by UNIV. Activated charcoal was purchased from Wako Pure Chemical Industries (Japan). Methanol was supplied by RANKEN. The methanol had a boiling point of 65°C, viscosity of 0.54, miscible with water and dielectric constant of 32.7. Acetonitrile was supplied by Avon Chem. It had a boiling point of 82°C, viscosity of 0.34 cp at 25°C, miscible with water at 20°C and dielectric constant of 37.5. Dichlorvos reference standards (98.0% purity) were obtained from Accu Standards. Shimadzu High Performance Liquid Chromatography-UV-VIS spectroscopy was procured from Kyoto, Japan. A standard curve was prepared from which by using the height and area under a curve the sample dichlorvos concentrations were determined. For data analysis a one way t-test was employed in which the average concentration of dichlorvos in each of the three vegetable types were compared to the standard MRL for any significant difference and significance was accepted when p< 0.05

## Results

### Levels of dichlorvos residues found in Lettuce from the study site

**Figure 1:**
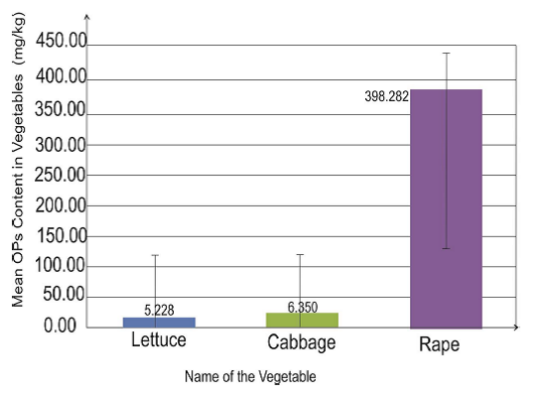
Mean dichlorvoslevels in lettuce, cabbage and rape

Out of the 14 lettuce samples tested 4(29%) of them did not have detectable dichlorvos residues, 2(14%) had residues levels below the MRL and 8 (57%) samples had dichlorvos residue above the safe limit. The mean dichlorvosresidue level in lettuce was 5.23mg/kg which was significantly higher than the MRL (p< 0.05) (Table 1) (Figure 1).

**Table 1:** Average dichlorvos levels in lettuce, cabbage, rape; and proportion of samples with undetectable dichlorvos, with detectable dichlorvos levels below MRL and above MRL

Vegetable	MRL(mg/kg)	Aver. Conc(mg/kg)	% undetectable	%	%	
				˂ MRL	˃ MRL	*p*
Lettuce	1 mg/Kg	5.228	29	14	57	˂0.05
Cabbage	1 mg/Kg	6.35	0	60	40	0.05
Rape	1 mg/Kg	398.282	0	0	100	˂0.05

### Levels of dichlorvos residues found in cabbage from the study site

This study revealed that out of the 15 cabbage samples 9(60%) of them had dichlorvos residues level below the maximum residue limit and 6 (40%) samples had dichlorvos residue above the MRLs. The mean dichlorvos residue level in cabbage was 6.35mg/kg which was higher than the MRL (p = 0.05) (Table 1) (Figure 1).

### Levels of dichlorvos residues found in rape from the study site

**Figure 2:**
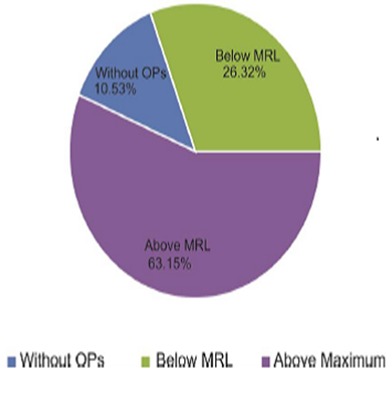
Overall percentage distribution of dichlorvos in all 38 vegetable samples

This study revealed that out of the 9 rape samples all of them had dichlorvos residues level above the maximum residue limit. The mean dichlorvos residue level in rape was 398.28mg/kg which was significantly higher than the MRL (p< 0.05)(Table 1) (Figure 1). This research also revealed that out of the 38 vegetables sampled,4 (11%) did not have detectable dichlorvos, 10 (26%)had dichlorvos residues below the MRL and 24(63%) had dichlorvos residues above the MRLs (Figure 2). The mean residue level in samples all 38 samples was found to be 98.76mg/kg which was significantly higher than the MRL (p< 0.05)

## Discussion

On average the dichlorvos residue levels that were recorded in the vegetables were higher than the Food and Drugs Act (Zambian), FAO and WHO recommended standard values. The highest mean dichlorvos level was in rape samples followed by cabbage and lettuce in decreasing order. This high level of dichlorvos could be due to the misapplication such as over applying the pesticides and the indiscriminate use of chemical pesticides to achieve higher vegetable yields. Also improper disposal of insecticide containers that bring about the liberation of these insecticide residues may be a contributing factor. This improper use of pesticides may lead to emergency of resistant pest populations, which would be insensitive to normal pesticide dosages [17, 18].

It must be underscored that illiteracy, poor or bad attitudes of farmers on dangers of pesticides could contribute to higher levels of dichlorvos in vegetables. For instance some farmers may be mixing one pesticide with the other in the pursuit of higher concentration of pesticide that is more effective in eradicating the pests. The higher levels of dichlorvos could be due to not following Good Agricultural Practices such as not following the recommended withdraw period, which is the minimum time one must wait between applying a pesticide and final harvesting of the crop or vegetable.

In a study done in Ghana which looked at a wide range of organophosphates in vegetables, dichlorvos was the most frequently detected residue in all the samples though at a much lower concentration than in samples we tested [19]. In Zambia, laws have been enacted and regulatory agencies have been established that regulate the importation of pesticides and monitor pesticide residues in the vegetables. However lack of effective implementation of these laws may contribute to higher levels of dichlorvos in the vegetables. The enforcement agencies are having to endure logistical constraints like poor funding, lack of transport and manpower. Furthermore, the cost of conducting pesticides residue monitoring is prohibitive for the local authorities to embark on. To make matters worse, these agencies have no clearly defined policy and programs on how and when the monitoring of the pesticide residue may be conducted in vegetables.

It is evident that there are high levels of dichlorvos in vegetables consumed in Zambia. It is highly likely that this could be due to not following proper methods of spraying vegetables, use of fault equipment and not following proper guidelines on when to consume vegetables that have just been sprayed [20]. It is therefore important that levels of dichlorvos are ascertained in order to protect human health due to cumulative effect of the pesticides [19, 21]. In addition pesticides like dichlorvos have also been shown to have a detrimental effect on soil micro flora and fauna. According to our results rape is a better accumulator of dichlorvos followed by cabbage and lettuce. Rape may apparently appear to be a better accumulator possibly because rape sells faster on the market and in order to increase profit margin farmers may be applying their insecticides on rape more aggressively. There is also a possibility that rape may be supplied on the market much earlier than the minimum time that is allowed between spraying and harvesting.

The clinical picture of toxicity to organophosphates in vegetables is different from that of acute exposure to high levels such as accidental inhalation where an individual may present with excessive salivation, lachrymation, urination, defecation, gastrointestinal distress and emesis [22]. The cumulative effect of dichlorvos has potential to gradually affect the health and wellbeing of consumers. Studies have shown that even low level exposure to organophosphates can cause neuropsychiatric conditions [23]. This though was not the case in one study where no association was observed between the behavioral score in children and exposure to low levels of organophosphates [24]. Perhaps even more worrying is the possible link between organophosphates and the development of obesity and type 2 diabetes mellitus as reported in one study [22].

Emphasis has been on diet, life style, and genetics as factors associated with obesity and type 2 diabetes mellitus but the possible role of organophosphates in etiology of the two conditions has not received much attention. Organophosphates primarily produce their insecticidal activity, as well as their systemic toxicity in humans, by inhibiting acetyl cholinesterase, the enzyme that breaks down the neurotransmitter, acetylcholine. In addition, organophosphates have also been shown to disrupt cell signaling cascades; among the important pathways is the cAMP-adenylyl cyclase signaling pathway [25–28]. As a result cellular responses to neurotransmitters, hormones, cytokines and signals that operate through cyclic AMP are permanently altered and this can disrupt metabolic, cardiovascular and hormonal responses which can possibly lead to metabolic syndrome. It is highly likely that nearly every person has organophosphate residues in their bodies and that most exposures are below that threshold and thus go unnoticed and undetected [29].

The increasing prevalence of obesity and type 2 diabetes especially in urban area may in part be due to pesticide residues on fruits and vegetable eaten. There has been a general decline in fertility in many countries as manifested by declining sperm counts. In one study which looked at the effect of food pesticide residues on sperm count it was found that there was a negative correlation between pesticide levels in semen and the sperm count [30], which suggests that pesticides may be responsible to some extent for the worldwide decline in fertility. Organophosphates have also been linked to malignancy, Parkinson's disease. Findings of a study in France suggested that there were neurologic manifestations in elderly persons who were exposed occupationally to pesticides [31]. Another study carried out in France showed that organophosphates in vineyards contributed to mortality from brain cancer among farmers [32]. The above is evidence that chronic exposure to pesticides can cause health problems, and unfortunately there is paucity of research data that clearly states the long term health effects to chronic bioaccumulation of dichlorvos in human body [21].

The study had a number of limitations which included inadequate funding which restricted coverage to four areas within Lusaka. Soil samples from the farms were not analyzed for the presence of dichlorvos due to prohibitive costs that were going to be incurred. This study only looked at dichlorvos and only three vegetables. Therefore it is highly recommended that another study be conducted that will include a variety of pesticides and a variety of fruits and vegetables. There should be a deliberate policy to educate farmers on the proper use and long term health effects of organophosphate insecticides.

## Conclusion

According to our findings out of the 38 vegetables sampled 63% had dichlorvos residues well above the safe limit, 26.% contained dichlorvos below the safety limit and 11% did not have any detectable dichlorvos residues. The average dichlorvos residue levels in the 38 samples was significantly above the maximum residue limit as set by the Zambian Food and Drugs Act.

### What is known about this topic

- There is wide spread unregulated use of dichlorvos in third world countries in an effort to increase food production.
- As long as the proper guidelines for use of dichlorvos are adhered to, exposure through food contamination can be significantly reduced
- Dichlorvos is a direct inhibitor of acetylcholinesterase and hence chronic exposure has potential for causing serious neural and muscular disorders.

### What this study adds

- There are unacceptably high levels of dichlorvos on commonly consumed vegetables in Lusaka, Zambia.
- There is need for a regulatory body to be monitoring dichlorvos levels in vegetables on a regular basis using robust guidelines.
- Public education should be strengthened at all levels from farmers to consumers on the dangers of dichlorvos to health.
